# The Interment of Dr. Solomon Smith

**Published:** 1903-04-18

**Authors:** 


### THE INTERMENT OF DR. SOLOMON SMITH.

### HIS WORK AT HALIFAX.

The funeral of the late Dr. Solomon C. Smith took place at Stoney-royd Cemetery, Halifax, on Thursday, April 9th. The ceremony was conducted by the Ven. Archdeacon Brooks, M.A., Vicar of Halifax, and the Rev. F. M. Sykes, B.A. Both at the service in the church and later at the cemetery there were present a large number of the relations and private friends of the deceased, and also representatives of the Halifax Municipality, the medical practitioners of Halifax, the Board of the Royal Infirmary, the Halifax Scientific Society, representatives of other local societies and associations, and Dr. Burrows, who represented Sir Henry Burdett, K.C.B., editor of The Hospital. The floral tributes were numerous and beautiful and amongst them were wreaths from Sir Henry Burdett and from the editorial staff of The Hospital.

Dr. Solomon Smith was born in Halifax, where his father was in medical practice, in 1842. He was educated at the Heath Grammar School, and afterwards received his medical training at Birmingham, London (Guy's Hospital), and Paris. In 1867 he succeeded to his father's practice in Halifax, where his sterling qualities and true worth were not long without recognition, and where he ultimately occupied a unique position. He became surgeon to the Halifax Infirmary, an institution whose welfare he materially advanced and to which he remained affectionately attached till the time of his death. The fine buildings at the present Royal Halifax Infirmary owe much to Dr. Smith's interest and practical knowledge. The institution is built on the arch system, of which it is an admirable example. This, and the general hygienic arrangements were advocated by Dr. Smith. He was the first to give lectures to the nurses at the infirmary, and he always took the keenest interest in all matters which affected in any way the prosperity or welfare of nurses generally; and latterly more especially of those working under the Poor Law, and for this reason he became a member of the executive committee of the Workhouse Nursing Association. Dr. Smith was a man of wide sympathies; he was President of the University Extension Association in Halifax, and many were the interesting lectures he gave before the Literary and Philosophical Society. Rather reserved and shy in his manner, it took some time to thoroughly get to know him, and only those who knew him well were aware of his kindness and the wonderful care that he gave to his patients. He was one of those men who lived entirely in his work, requiring no amusements and but little relaxation. During the 25 years that he lived and practised in Halifax he established a place in the hearts of those with whom he was in any way associated which the passing of time will not eradicate. On his leaving Halifax in 1892 for London Dr. Smith was the recipient of a handsome testimonial at the hands of the townspeople. This testimonial consisted of a portrait in oils of himself (which hangs in the public library in the town) and a silver tea-service. The speeches which were made at the time of the presentation testify to the great esteem in which he was held. He had an extraordinary power of illustrating scientific subjects, and was of an original and inventive turn of mind. An old friend of his recalls the fact that he was one of the few people who expressed pleasure at the visit of plumbers to his house, because they required (in his own striking phrase) so much "adjustment to difficulty."

